# CYP1B1: A Novel Molecular Biomarker Predicts Molecular Subtype, Tumor Microenvironment, and Immune Response in 33 Cancers

**DOI:** 10.3390/cancers14225641

**Published:** 2022-11-17

**Authors:** Benchao Yuan, Guihong Liu, Zili Dai, Li Wang, Baisheng Lin, Jian Zhang

**Affiliations:** 1 Department of Oncology and Hematology, The Sixth People’s Hospital of Huizhou City, Huiyang Hospital Affiliated to Southern Medical University, Huizhou 516003, China; 2 Department of Radiation Oncology, Dongguan Tungwah Hospital, Dongguan 523120, China; 3 Department of Radiation Oncology, Affiliated Cancer Hospital & Institute of Guangzhou Medical University, State Key Laboratory of Respiratory Diseases, Guangzhou Institute of Respiratory Disease, Guangzhou 510095, China; 4 Guangzhou Medical University, Guangzhou 511495, China

**Keywords:** pan-cancer, CYP1B1, tumor mutation burden, microsatellite instability, neoantigen, immune activity

## Abstract

**Simple Summary:**

Cytochrome P450 Family 1 Subfamily B Member 1 (CYP1B1) is a critical metabolic enzyme of melatonin. Although melatonin has been identified to exhibit tumor suppressing activity, the role and mechanism of the clinical and immunological characteristics of CYP1B1 in cancer remain unclear. We comprehensively explored the clinical and immunological characteristics of CYP1B1. We identified that the dysregulated expression of CYP1B1 was associated with clinical characteristics and a tumor immune microenvironment, which may provide a promising predictor and molecular target for clinical immune treatment.

**Abstract:**

Background: Cytochrome P450 Family 1 Subfamily B Member 1 (CYP1B1) is a critical metabolic enzyme of melatonin. Although melatonin has been identified to exhibit tumor suppressing activity, the role and mechanism of the clinical and immunological characteristics of CYP1B1 in cancer remain unclear. Methods: In this study, RNA expression and clinical data were obtained from The Cancer Genome Atlas (TCGA) across 33 solid tumors. The expression, survival, immune subtype, molecular subtype, tumor mutation burden (TMB), microsatellite instability (MSI), biological pathways, and function in vitro and vivo were evaluated. The predictive value of CYP1B1 in immune cohorts was further explored. Results: We found the dysregulated expression of CYP1B1 was associated with the clinical stage and tumor grade. Immunological correlation analysis showed CYP1B1 was positively correlated with the infiltration of lymphocyte, immunomodulator, chemokine, receptor, and cancer-associated fibroblasts (CAFs) in most cancer. Meanwhile, CYP1B1 was involved in immune subtype and molecular subtype, and was connected with TMB, MSI, neoantigen, the activation of multiple melatonergic and immune-related pathways, and therapeutic resistance. Conclusions: Together, this study comprehensively revealed the role and mechanism of CYP1B1 and explored the significant association between CYP1B1 expression and immune activity. These findings provide a promising predictor and molecular target for clinical immune treatment.

## 1. Introduction

Melatonin, an endogenous hormone, is secreted by the pineal gland in response to biological rhythm [1,2]. The synthesis and metabolism of the hormone involves a series of biological pathways. In the process of melatonin synthesis, N-acetylserotonin (NAS) was firstly combined to serotonin by arylalkylamine N-acetyltransferase (AANAT) and methylated by acetylserotonin O-methyltransferase (ASMT) [3]. The melatonin is further metabolized into 6-hydroxymelatonin (6OH-MEL) by human cytochrome P450 family [4]. Evidence shows that various biological properties of melatonin, such as the circadian clock [5,6,7], sleep regulation [8,9], anti-inflammatory properties [10,11,12], immune modulation [13,14,15], and anti-cancer activities [16,17,18], have been well revealed. Nevertheless, the clinical and immunological function of the cytochrome P450 family in cancer remains unclear.

Cytochrome P450 1B1 (CYP1B1), one member of the cytochrome P450 family, is an extrahepatic enzyme that is involved in the metabolism of melatonin [4] and other compounds [19,20,21,22]. The exogenous carcinogens, such as aromatic amines and polycyclic aromatic hydrocarbons, can be oxidized by CYP1B1 to active carcinogenic products. Accumulated evidence has revealed that the single nucleotide polymorphism of CYP1B1 is associated with the risk of cancer, including prostate, endometrial, and ovarian cancer [23,24,25]. Recent studies have also linked CYP1B1 expression to clinical grade lymphovascular invasion and lymph node metastasis [26,27]. CYP1B1 is also involved in the positive regulation of inflammatory cytokine, which acts on both cancer cells and the tumor microenvironment [28]. However, the role and mechanism of CYP1B1 in the immune microenvironment still remains to be demonstrated.

In this study, we investigated the clinical and immunological aspects of the metabolic enzyme CYP1B1 among 33 solid tumors from The Cancer Genome Atlas (TCGA) databases. We identified that the dysregulated expression of CYP1B1 was associated with clinical stage, grade, and survival. CYP1B1 was involved in the infiltration of the lymphocyte, immunomodulator, chemokine, receptor, and cancer associated fibroblast (CAF) in cancer. Furthermore, we also probed insight into the immune subtype, molecular subtype, tumor mutation burden (TMB), microsatellite instability (MSI), neoantigen, and immune-related pathways mediated by CYP1B1, which may provide a promising predictor and molecular target for clinical immune treatment.

## 2. Materials and Methods

### 2.1. Data Collection

The expression, phenotype, and survival data were obtained from the UCSC Xena database (https://xenabrowser.net/, accessed on 22 May 2020). The lymphocyte, immunomodulator, chemokine, immune subtype, and molecular subtype were downloaded from the Tumor-Immune System Interactions (TISIDB) database (http://cis.hku.hk/TISIDB/index.php, accessed on 23 March 2019). The cancer-associated fibroblasts (CAFs) data were obtained from TIMER 2.0 (http://timer.cistrome.org/, accessed on 2 July 2020). The TCGA database was used to obtain the tumor mutation burden (TMB), microsatellite instability (MSI), and neoantigen. The study was conducted in accordance with the Declaration of Helsinki (as revised in 2013) and was approved by the Ethics Committee of The Sixth People’s Hospital of Huizhou City (PJ2022MI-KJ038).

### 2.2. Differential Gene Expression Analysis

To identify the expression differences of CYP1B1 between tumor samples and normal tissues, the expression data of 33 cancers was downloaded from the UCSC Xena database. The expression values were normalized by Transcripts Per Million (TPM) transformation. A distinction with a threshold of *p* < 0.05 was considered as having a significance.

### 2.3. Survival Analysis

The KM-plotter analysis of 33 cancer patients were examined using univariate COX regression analysis to determine the prognostic significance of CYP1B1. The forest plot was performed using the R software forest plot package. A log-rank test with a threshold of *p* < 0.05 was considered as having a significance.

### 2.4. Correlation Analysis between CYP1B1 and Tumor Immune System

To clarify the relation between CYP1B1 and the tumor immune system, the potential relationship among CYP1B1 and 28 lymphocytes, 69 immunomodulators (45 immunostimulators, 24 immunoinhibitors), 41 chemokines, and 18 receptors, immune subtypes and molecular subtypes were explored using the TISIDB database. The relationship of CYP1B1 and CAF was assessed by the TIMER 2.0 database. The correlation of CYP1B1 and TMB, MSI, and neoantigen were further evaluated. *p* < 0.05 was considered as having a significance.

### 2.5. Single-Sample Gene Set Enrichment Analysis (ssGSEA)

To identify the CYP1B1 activity in cancer, the single sample gene sets enrichment analysis (ssGSEA) between high CYP1B1 and low CYP1B1 expression was evaluated by using R software GSVA package. *p* < 0.05 was considered as having a significance.

### 2.6. Association between CYP1B1 and Drug Response

CCLE gene expression data were quantile normalized among all different cell lines for partial correlation, and then Z-score normalization was applied in each tissue to calculate the expression difference between High–Low (using the median as a cutoff) IC50 groups. The X-axis indicates the mean/median expression difference across tissues. Correlations of CYP1B1–drug associations after controlling for the tissue average expression were analyzed.

### 2.7. Gene Set Enrichment Analysis (GSEA)

Correlations with other genes and ordered genes based on findings were performed to discover the biological aspects of CYP1B1. The sorted gene list was used in GSEA analysis to see if highly linked genes clustered in genuinely functional pathways. The reference gene set was annotated gene set c5.go.v7.4.symbols.gmt and c2.cp.kegg.v7.4.symbols.gmt. As previously stated, FDRs of 0.05 and *p*-values of 0.01 were considered significant.

### 2.8. Cell Culture

The lung cancer cell line PC9, breast cancer cell lines MDA-MB-231 was obtained from the Sun Yat-sen University Cancer Center. Cells were maintained at 37°, 5% CO_2_ in 10% DMEM (Invitrogen, Carlsbad, CA, USA) or RPMI-1640 (Thermo Fisher Scientific, Waltham, MA, USA) supplemented with 10% fetal bovine serum.

### 2.9. Wound Healing Assay

Cells were cultured in 10% DMEM (Invitrogen, Carlsbad, CA, USA) or RPMI-1640 (Gibco, Australia), supplemented with 10% fetal bovine serum at 37°, 5% CO_2_. The medium was removed, and the surface of the inoculated cells was scratched and marked with a 10 μL pipette tip. A sterile 200-L pipette tip was used to scrape the surface of the cell monolayer to create the wound. Under an inverted microscope, photographs were taken at time 0 and 24 h after cell scratching (Olympus Corporation). The area of each wound was calculated using Image J software (National Institutes of Health).

### 2.10. Transwell Assay

Transwell plates (8-m pores) were used for Transwell migration or invasion studies. Next, 5 × 10^4^ (migration assay) or 1 × 10^5^ (invasion assay) cells resuspended in serum-free medium were placed in the top chamber, either uncoated or covered with Matrigel (BD Biosciences). The culture medium in the lower compartment included 10% FBS. The cells were fixed and stained after being incubated for 12 or 24 h. Cells on the undersides of the filters were seen and counted at a magnification of 200 magnification.

### 2.11. Cell Proliferation Assay

The cancer cell line was seeded in 1000 cells per plate and cultured for two weeks at 37 °C in a 5% CO_2_ incubator. The cells were washed twice, fixed for 15 min with 4% paraformaldehyde, then stained for 20 min at room temperature with 1% crystal violet solution. The number of visible colonies was determined.

### 2.12. In Vivo Metastasis Model

The Guangzhou Medical University Cancer Center’s Institutional Animal Care and Use Committee authorized the animal operations (Guangzhou, China, G2022-050). First, 1 × 10^7^ PC9 cells were injected into the footpads of mice for the tumor metastasis model. After 2 weeks, the mice were euthanized, and their footpad tumors and inguinal lymph nodes were removed after being intraperitoneally administered with melatonin (25 mg/kg) every other day for 7 consecutive days.

### 2.13. Immunohistochemistry Analysis

The deparaffinized sections were incubated with 5% normal goat serum (Beyotime, Shanghai, China) to block endogenous peroxidase activity, then the tumor sections were incubated with the primary anti-CYP1B1 (Proteintech, Wuhan, China, 18505-1-AP, 1:200), anti-CD31 (Abcam, Cambridge, UK, ab182981, 1:2000), Ki67 (Abcam, ab16667, 1:400), LY6G (Abcam, ab238132, 1:1000), MMP9 (Boster, Wuhan, China, PB9669, 1:200) antibody at 4 °C overnight. After incubation with the secondary antibody, the staining was visualized using the DAKO REAL EnVision Inspection System (DAKO).

### 2.14. Statistical Analysis

All statistical analyses were performed using R software (version 4.0.3). The correlations between CYP1B1 and clinicopathological features were detected by Chi square test. Univariate analysis was used to estimate the prognostic value of CYP1B1. The correlation analysis was assessed by the Spearman rank test. *p* < 0.05 was considered statistically significant.

## 3. Results

### 3.1. The Expression, Tumor Stage and Clinical Grade of CYP1B1 in Cancer

**Figure 1:**
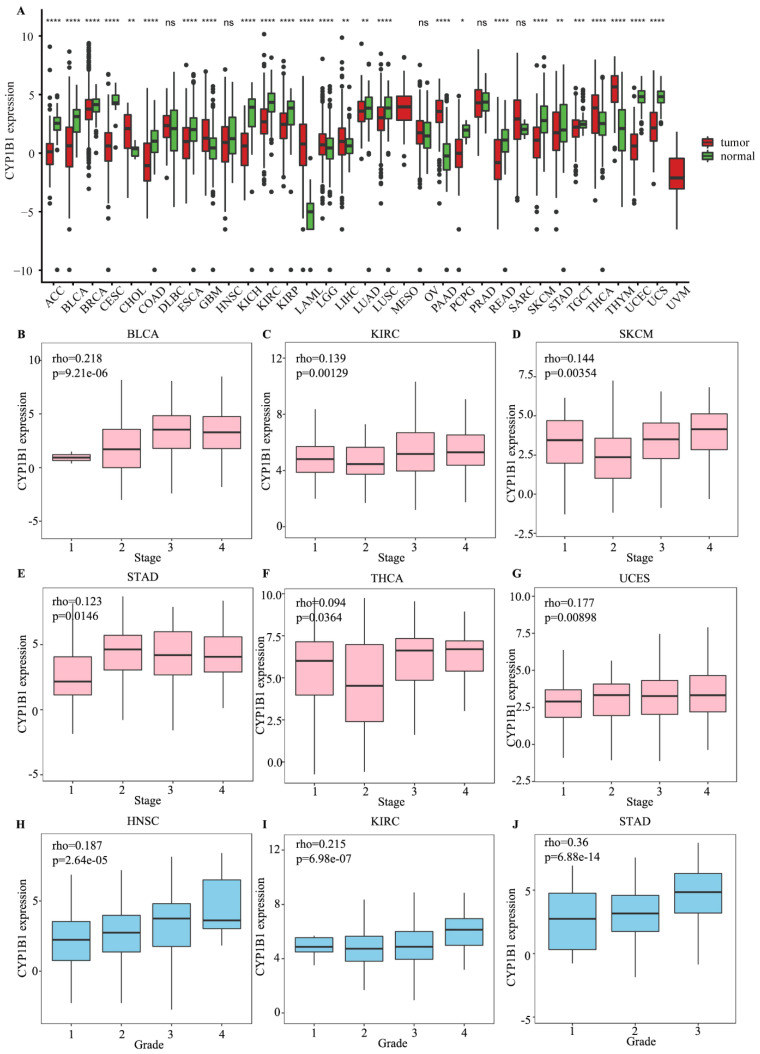
CYP1B1 expression, stage and grade in cancer. (**A**) CYP1B1 expression between tumor and normal samples in cancer. (**B**–**G**) The positive correlation between CYP1B1 expression and clinical stage in in BLCA (**B**), KIRC (**C**), SKCM (**D**), STAD (**E**), THCA, (**F**) and UCEC (**G**). (**H**–**J**) The positive correlation between CYP1B1 expression and tumor grade in HNSC (**H**), KIRC (**I**), and STAD (**J**). * means *p* < 0.05; ** means *p* < 0.01; *** means *p* < 0.001; **** means *p* < 0.0001; *ns* means no significance.

By investigating RNA-seq data from 33 cancers in the TCGA, we explored the differential expression of CYP1B1 between tumor samples and healthy samples. As shown in Figure 1A, CYP1B1 was significantly downregulated in most of the cancers. 

The relationship between CYP1B1 expression and clinical prognosis in 33 cancers was further analyzed. According to the median expression of CYP1B1, the cancers were divided into high- and low-expression groups. CYP1B1 was associated with protective overall survival (OS) in SKCM and SARC, and risky OS in STAD, KIRC, and BLCA (Figure S1A). Similarly, CYP1B1 was associated with protective disease-specific survival (DSS) in THYM and disease free interval (DFI) in LGG (Figure S1B,C). CYP1B1 was associated with risky DSS in BLCA, COAD, KIRC, KIRP and STAD, DFI in OV and STAD, and progression free interval (PFI) in GBM, KIRC, and STAD (Figure S1B–D). The associations between CYP1B1 and the clinical stage showed that a higher expression of CYP1B1 was positively related to the tumor stage in BLCA (r = 0.218, *p* = 9.21 × 10^−6^; Figure 1B), KIRC (r = 0.139, *p* = 0.00129; Figure 1C), SKCM (r = 0.144, *p* = 0.00354; Figure 1D), STAD (r = 0.123, *p* = 0.0146; Figure 1E), THCA (r = 0.094, *p* = 0.0364; Figure 1F), and UCEC (r = 0.177, *p* = 0.00898; Figure 1G). The associations in the tumor grade found that the lymphocyte, immunomodulator, chemokine, and receptor were positively related to the tumor grade in HNSC (r = 0.187, *p* = 2.64 × 10^−5^; Figure 1H), KIRC (r = 0.215, *p* = 6.98 × 10^−7^; Figure 1I), STAD (r = 0.123, *p* = 0.0146; Figure 1J), and negatively related with grade in LIHC (r = −0.159, *p* = 0.00224; Figure S2). These findings suggested that dysregulated CYP1B1 expression might serve as a predictive biomarker for cancer.

### 3.2. The Correlation among CYP1B1 Expression and Lymphocyte, Immunomodulator, Chemokine and Receptor

**Figure 2:**
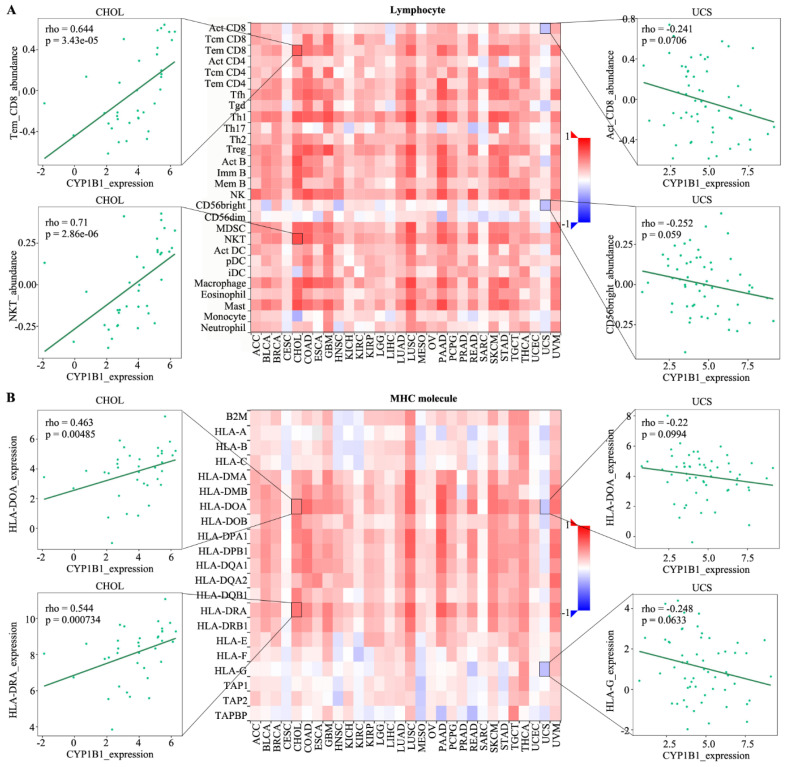
The correlations between CYP1B1 expression and lymphocyte and MHC molecule. (**A**) The spearman correlations between CYP1B1 expression and lymphocyte in cancer. (**B**) The spearman correlations between CYP1B1 expression and MHC molecule in cancer. The heatmap represents rho value. Red color means positive correlation, blue color means negative correlation. Four associations were showed by dot plots.

To identify the function of CYP1B1 expression in immune regulation, CYP1B1 expression was positively associated with lymphocyte and MHC molecule in most cancer. As shown in Figure 2A, CYP1B1 was positively related to Tem CD8 cell (r = 0.644, *p* = 3.43 × 10^−5^) and NKT cell (r = 0.71, *p* = 2.86 × 10^−6^) in CHOL and negatively related to act CD8 cell (r = −0.241, *p* = 0.0706) and CD56 bright (r = −0.252, *p* = 0.059) in UCS. Regarding the MHC molecule, CYP1B1 was also positively related to HLA-DOA expression (r = 0.463, *p* = 0.00485) and HLA-DRA expression (r = 0.544, *p* = 0.000734) in CHOL, and negatively related to HLA-DOA expression (r = −0.22, *p* = 0.0994) and HLA-G expression (r = −0.248, *p* = 0.0633) in UCS (Figure 2B). 

Next, the role of CYP1B1 in the immunostimulator was further evaluated. As depicted in Figure S3A, CYP1B1 was positively related to CD40LG expression (r = 0.677, *p* = 1.0 × 10^−5^) and TNFSF13B expression (r = 0.536, *p* = 9.08 × 10^−4^) in CHOL and negatively related to act CD27 expression (r = −0.337, *p* = 0.0106) and KLRK1 expression (r = −0.274, *p* = 0.0392) in UCS. Further, regarding immunoinhibitor, CYP1B1 was positively associated with BTLA expression (r = 0.66, *p* = 1.94 × 10^−5^) and PDCD1LG2 expression (r = 0.628, *p* = 6.11 × 10^−5^) in CHOL and negatively associated with act CD160 expression (r = −0.273, *p* = 0.0402) and LAG expression (r = −0.294, *p* = 0.0268) in UCS (Figure S3B). 

In addition, considering the chemokine, CYP1B1 was positively associated with CCL14 expression (r = 0.659, *p* = 1.98 × 10^−5^) and CCL19 expression (r = 0.75, *p* = 7.51 × 10^−7^) in CHOL and negatively associated with act CCL20 expression in UCS (r = −0.396, *p* < 2.2 × 10^−16^) and CXCL1 expression (r = −0.424, *p* < 2.2 × 10^−16^) in STAD (Figure S4A); regarding the receptor, CYP1B1 was positively associated with CCR4 expression (r = 0.719, *p* = 2.03 × 10^−6^) and CXCR4 expression (r = 0.618, *p* = 8.53 × 10^−5^) in CHOL and negatively associated with act CCR6 expression in READ (r = −0.259, *p* = 7.46 × 10^−4^) and CCR9 expression (r = −0.461, *p* = 1.15 × 10^−4^) in KICH (Figure S4B). 

### 3.3. Correlation between CYP1B1 and CAFs in the Tumor Microenvironment

**Figure 3:**
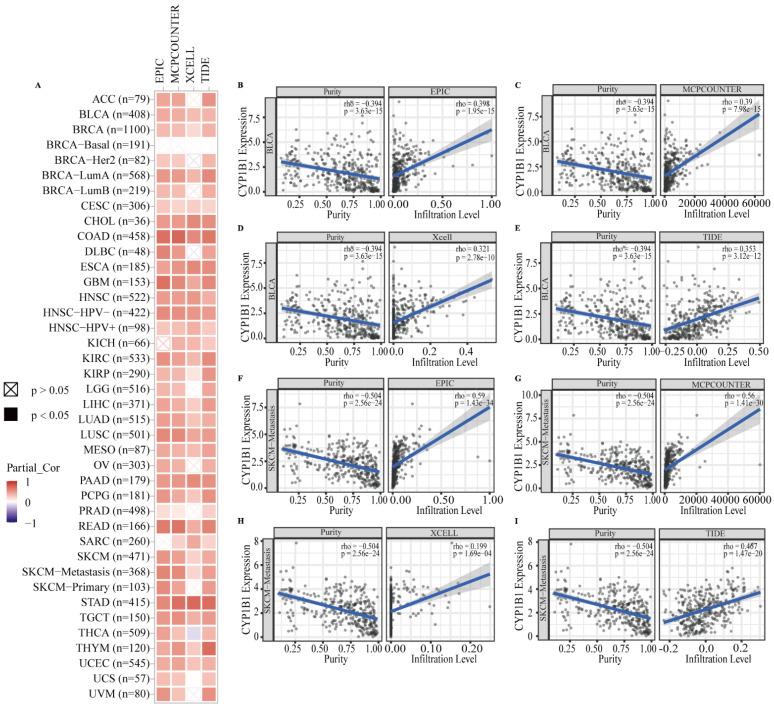
The correlations between CYP1B1 expression and immune infiltration of cancer-associated fibroblasts. (**A**) The correlation between CYP1B1 expression and CAFs infiltration estimated by the EPIC, MCPCOUNTER, XCELL, and TIDE algorithm in cancer. (**B**–**E**) The correlation between CYP1B1 expression and CAFs infiltration estimated by the EPIC (**B**), MCPCOUNTER (**C**), XCELL (**D**), and TIDE (**E**) algorithm in BRCA. (**F**–**I**) The correlation between CYP1B1 expression and CAFs infiltration estimated by the EPIC (**F**), MCPCOUNTER (**G**), XCELL (**H**), and TIDE (**I**) algorithm in SKCM-Metastasis.

CAFs, the most abundant stromal cells in the tumor microenvironment (TME), are associated with tumor cell growth, invasion, and metastasis, metabolic reprograming, immune escape, and therapeutic resistance [29,30,31]. Four algorithms, including EPIC, MCPCOUNTER, XCELL, and TIDE, were used to evaluate the correlation between the CAFs and the CYP1B1 expression level in 33 cancers. Cancers with consistent correlations of four algorithms were considered to be importantly associated with CAFs infiltration. As shown in Figure 3A, the expression of CYP1B1 was importantly positively correlated with CAFs infiltration in BLCA, BRCA, BRCA-LumA, CESC, CHOL, COAD, ESCA, GBM, HNSC, HNSC-HPV-, HNSC-HPV+, KIRC, KIRP, LIHC, LUAD, LUSC, MESO, PAAD, PCPG, READ, SKCM, SKCM-Metastasis, STAD, TGCT, THYM, and UCEC. The correlation estimated by the EPIC, MCPCOUNTER, XCELL, and TIDE algorithms were displayed as examples in Figure 3. For example, the expression of CYP1B1 was positively correlated with the level of infiltration of CAFs in BRCA (r = 0.398, *p* = 1.95 × 10^−15^; r = 0.39, *p* = 7.98 × 10^−15^; r = 0.321, *p* = 2.78 × 10^−10^; r = 0.353, *p* = 3.12 × 10^−12^; Figure 3B–E) and SKCM-Metastasis (r = 0.59, *p* = 1.43 × 10^−34^; r = 0.56, *p* = 1.41 × 10^−30^; r = 0.199, *p* = 1.69 × 10^−4^; r = 0.467, *p* = 1.47 × 10^−20^; Figure 3F–I). These results indicated that CAFs infiltration mediated by CYP1B1 were critical for cancer occurrence and progression in the TME.

### 3.4. Correlation between CYP1B1 and TMB, MSI and Neoantigen

**Figure 4:**
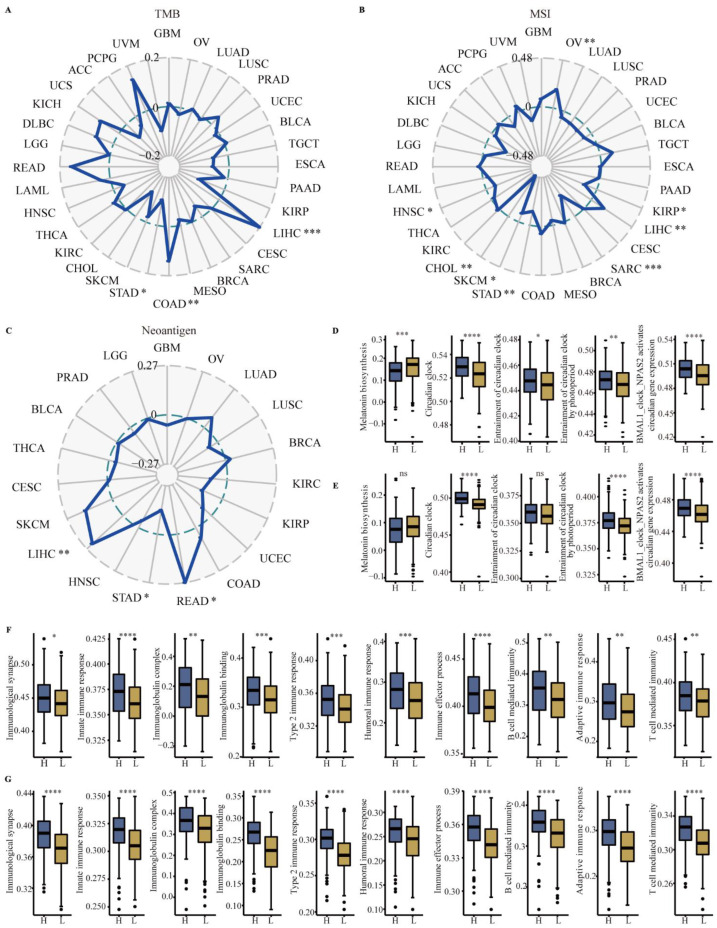
The correlations between CYP1B1 expression and TMB, MSI, neoantigen, and immune-related pathways. (**A**) Correlations between CYP1B1 expression and TMB in cancer. (**B**) Correlation between CYP1B1 and MSI in cancer. (**C**) Correlation between CYP1B1 and neoantigen in cancer. (**D**,**E**) The melatonin-related pathways enriched by ssGSEA analysis in LIHC (**D**) and STAD (**E**). (**F**,**G**) The classical immune related pathways enriched by ssGSEA analysis in LIHC (**F**) and STAD (**G**). Two groups (High-expression and Low-expression) of pathway scores with boxplots using the Mann-Whitney U test. * *p* < 0.05; ** *p* < 0.01; *** *p* < 0.001; **** *p* < 0.0001.

To understand the role of CYP1B1 in immunotherapy, the association between CYP1B1 and immunotherapy-related biomarkers (TMB, MSI and neoantigen) was further assessed. As shown in Figure 4, CYP1B1 expression was positively associated with the TMB in COAD (*p* = 0.0044; Figure 4A) and LIHC (*p* = 0.00011; Figure 4A), and a negative association was found in STAD (*p* = 0.038; Figure 4A). CYP1B1 expression positively correlated significantly with MSI in OV (*p* = 0.0019) and LIHC (*p* = 0.0038) in Figure 4B, while a negative association in KIRP (*p* = 0.03), SARC (*p* = 8.7 × 10^−5^), STAD (*p* = 0.0074), SKCM (*p* = 0.05), CHOL (*p* = 0.0032) and HNSC (*p* = 0.029) was identified in Figure 4B. Similarly, CYP1B1 expression positively correlated significantly with neoantigen in LIHC (*p* = 0.0012) and READ (*p* = 0.046) in Figure 4C, while a negative association in STAD (*p* = 0.037) was revealed in Figure 4C. The results indicated that immunotherapy-related biomarkers mediated by CYP1B1 may play important roles in immune pathways. As the CYP1B1 was one of metabolic enzymes in melatonin, we further explored the melatonin-related pathways, including melatonin biosynthesis, circadian clock, entrainment of the circadian clock, entrainment of the circadian clock by photoperiod, and BMAL1_clock_NPAS2 circadian gene expression. As shown in Figure 4D and E, a high expression of CYP1B1 was associated with the inactivation of melatonin biosynthesis in LIHC (*p* < 0.0001) and STAD, associated with the activation of the circadian clock, entrainment of the circadian clock, entrainment of the circadian clock by photoperiod, and BMAL1_clock_NPAS2 circadian gene expression (Figure 4D,E). These results indicated that melatonin metabolism mediated by CYP1B1 may be involved in the complexity and heterogeneity of TME in cancer. 

To further uncover the role of CYP1B1 in TME, 10 classical immune-related pathways, including immunological synapse, innate immune response, immunoglobulin complex, immunoglobulin binding, type 2 response, humoral immune response, immune effector process, B cell mediated immunity, adaptive immune response, and T cell mediated immunity, were enriched by ssGSEA analysis. Compared with low expression of CYP1B1, a high expression of CYP1B1 was significantly associated with the activation of immune-related pathways in LIHC (Figure 4F) and STAD (Figure 4G).

### 3.5. The Association of CYP1B1 Expression and Therapeutic Response

**Figure 5:**
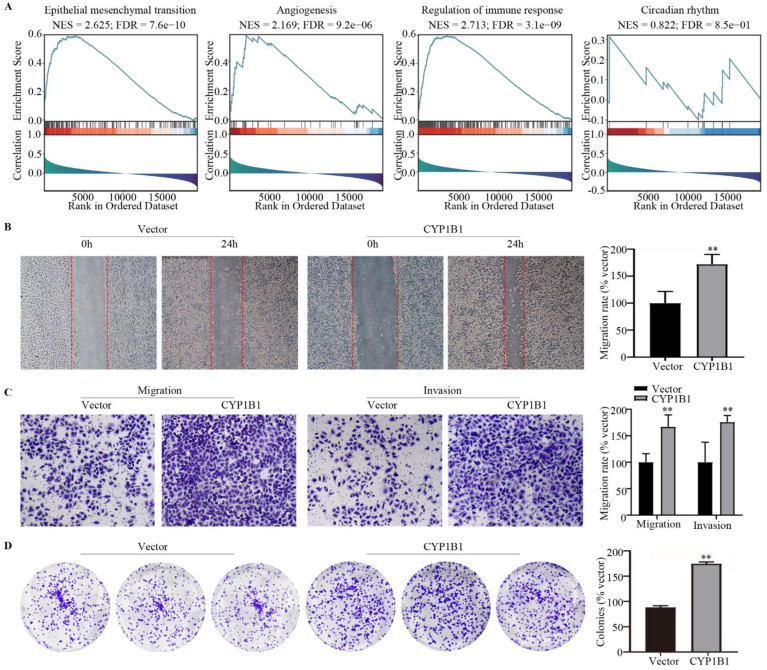
The function of CYP1B1 in vitro. (**A**) GSEA analysis of CYP1B1 in breast cancer. (**B**) Wound healing of CYP1B1. (**C**) Transwell migration and invasion assay of CYP1B1. (**D**) Colonies assay of CYP1B1. ** *p* < 0.01.

**Figure 6:**
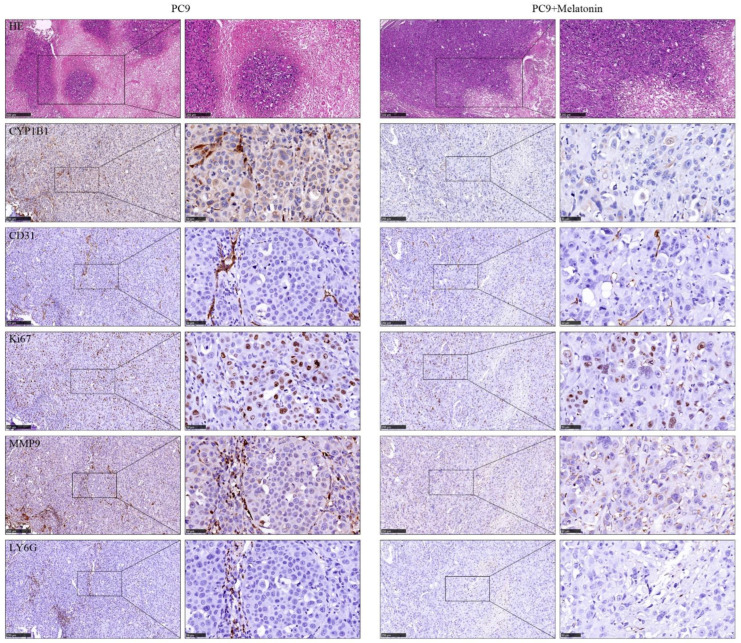
The HE and immunohistochemical staining of CD31, ki67, MMP9, and LY6G between PC9 and PC9 + Melatonin groups.

To identification the function of CYP1B1 in breast cancer, as shown in Figure 5A, GSEA analysis indicated that CYP1B1 was significantly associated with epithelial mesenchymal transition (NES = 2.625, FDR = 7.6 × 10^−10^), angiogenesis (NES = 2.169, FDR = 9.2 × 10^−6^), and regulation of the immune response (NES = 2.713, FDR = 3.1 × 10^−9^) and circadian rhythm (NES = 0.822, FDR = 8.5 × 10^−1^). Wound healing and transwell migration and invasion assays revealed that the overexpression CYP1B1 increased breast cancer migration and invasion (Figure 5B and C). Colony assays identified that CYP1B1 also improved breast cancer proliferation (Figure 5D). To explore the function of CYP1B1 in vivo, a tumor metastasis model was established and the immunohistochemical staining analysis of serial tissue sections showed that the expression of angiogenesis marker CD31, proliferation marker ki67, metastasis marker MMP9, and neutrophil infiltration marker LY6G mediated by CYP1B1 could be reversed by melatonin (Figure 6). 

### 3.6. Identification of Immune Subtype, Molecular Subtype and Immune Response

**Figure 7:**
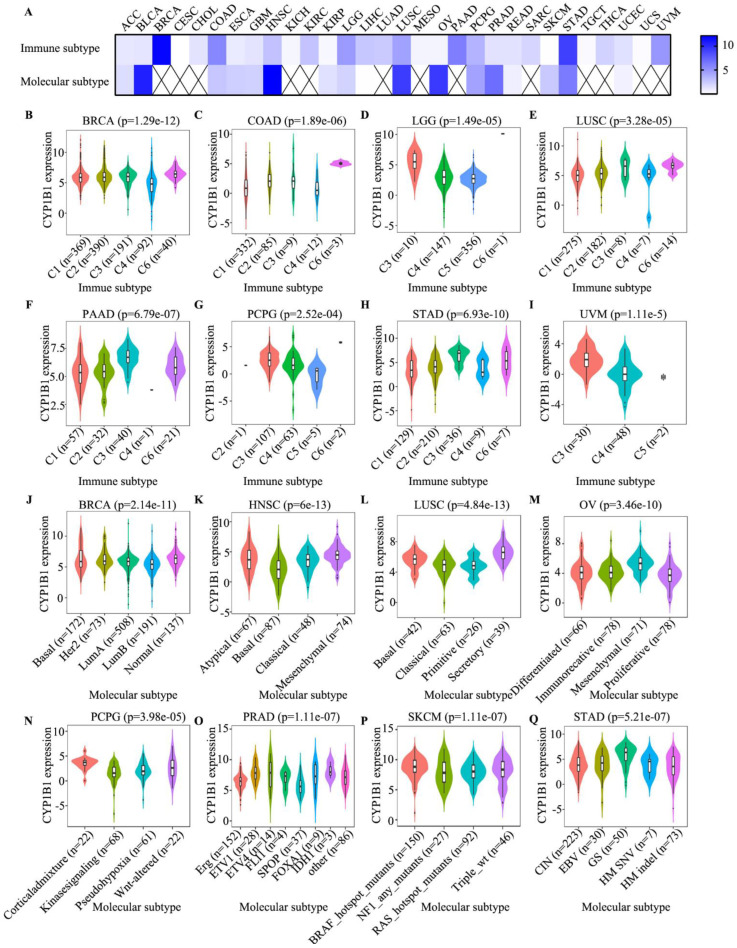
The associations between CYP1B1 expression and immune subtype and molecular subtype. (**A**) The heatmap of the Kruskal–Wallis test in the immune subtype and molecular subtype. (**B**–**I**) The top eight correlations between CYP1B1 expression and immune subtype in BRCA (**B**), COAD (**C**), LGG (**D**), LUSC (**E**), PAAD (**F**), PCPG (**G**), STAD (**H**), and UVM (**I**). (**J**–**Q**) The top eight correlations between CYP1B1 expression and molecular subtype in BRCA (**J**), HNSC (**K**), LUSC (**L**), OV (**M**), PCPG (**N**), PRAD (**O**), SKCM (**P**), and STAD (**Q**) (Kruskal–Wallis test, *p* < 0.05 was considered to be significant.).

The associations of CYP1B1 expression on immune and molecular subtypes in cancers was investigated. Overall, six immune subtypes, including wound healing (C1 subtype), IFN-γ dominant (C2 subtype), inflammatory (C3 subtype), lymphocyte depleted (C4 subtype), immunologically quiet (C5 subtype), and TGF-β dominant (C6 subtype) were analyzed. As depicted in Figure 7A, the expression of CYP1B1 was significantly associated with immune subtype in BRCA, CHOL, COAD, HNSC, KIRC, LGG, LIHC, LUAD, LUSC, PAAD, PCPG, PRAD, READ, SARC, STAD, THCA, UCEC, and UVM. The top eight immune subtypes, including BRCA (*p* = 1.29 × 10^−12^; Figure 7B), COAD (*p* = 1.89 × 10^−6^; Figure 7C), LGG (*p* = 1.49 × 10^−5^; Figure 7D), LUSC (*p* = 3.28 × 10^−5^; Figure 7E), PAAD (*p* = 6.79 × 10^−7^; Figure 7F), PCPG (*p* = 2.52 × 10^−4^; Figure 7G), STAD (*p* = 6.93 × 10^−10^; Figure 7H), and UVM (*p* = 1.11 × 10^−5^; Figure 7I), were revealed. 

The associations between CYP1B1 expression and molecular subtypes were also significantly clarified in ACC, BRCA, COAD, ESCA, GBM, HNSC, KIRP, LGG, LUSC, OV, PCPG, PRAD, SKCM, and STAD. The top eight molecular subtypes, including BRCA (*p* = 2.14 × 10^−11^; Figure 7J), HNSC (*p* = 6 × 10^−13^; Figure 7K), LUSC (*p* = 4.84 × 10^−13^; Figure 7L), OV (*p* = 3.46e-10; Figure 7M), PCPG (*p* = 3.98e-05; Figure 7N), PRAD (*p* = 1.11 × 10^−7^; Figure 7O), SKCM (*p* = 1.11 × 10^−7^; Figure 7P), and STAD (*p* = 5.21 × 10^−7^; Figure 7Q), were also identified. These results indicated that the dysregulated expression of CYP1B1 was associated with different immune subtypes and molecular subtypes in cancer.

**Figure 8:**
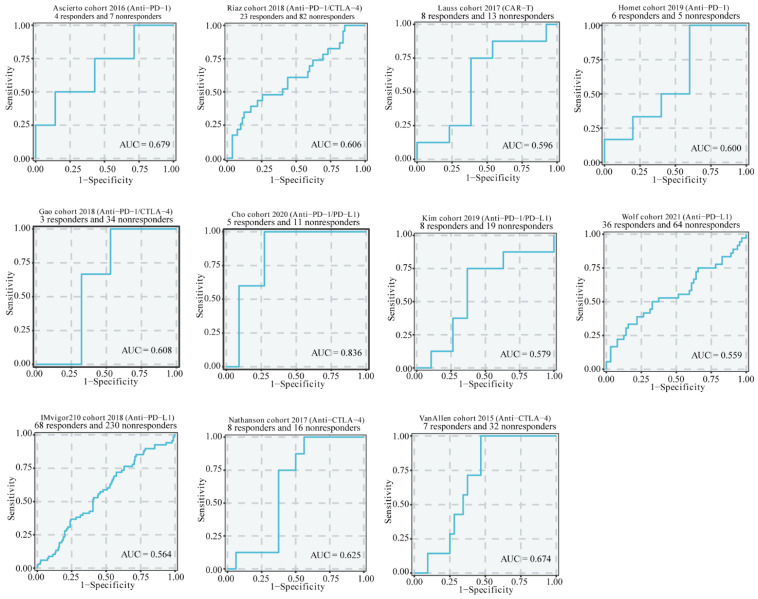
The predictive power of CYP1B1 in GSE67501, GSE91061, GSE100797, GSE111636, GSE115821, GSE126044, GSE135222, GSE173839, IMvigor210, Nathanson cohort 2017, VanAllen cohort 2015 immune cohorts.

To further reveal the clinical application of CYP1B1, we first evaluate the roles of CYP1B1 in GSE67501, GSE91061, GSE100797, GSE111636, GSE115821, GSE126044, GSE135222, GSE173839, IMvigor210, Nathanson cohort 2017, VanAllen cohort 2015, and immune cohorts. The predictive power of CYP1B1 was 0.679, 0.606, 0.596, 0.600, 0.608, 0.836, 0.579, 0.559, 0.564, 0.625, and 0.674, respectively, in anti-PD-1/PD-L1/CAR-T/CTLA4 cohorts (Figure 8). As shown in Figure S5, IC50 of HDAC, TOP1, RAF, c-MET, MEK, RAF, CDK4, ALK, RTK, GS, FGFR, and MDM2 inhibitor in high CYP1B1 expression was significantly upregulated, which indicated that CYP1B1 was associated with therapeutic resistance. However, the expression of CYP1B1 was also associated with the therapeutic sensitivity of AZD530 and erlotinib (Figure S5, *p* < 0.05). These results revealed that CYP1B1 may be a promising molecular target in clinical treatment.

## 4. Discussion

Melatonin has been considered a promising anti-cancer drug in the treatment of cancer [32,33,34]. However, the molecular and clinical characteristics of the melatonergic metabolic enzyme CYP1B1 in cancer still remain unknown. In this study, we comprehensively investigated the clinical and immunological pattern of CYP1B1 determined from RNA-seq data across TCGA pan-cancer. The results indicated that the dysregulated expression of CYP1B1 was correlated with clinical and immunological characteristics in cancer and could be a promising predictor and molecular target for clinical immune treatment.

CYP1B1, one member of the cytochrome P450 family, has been identified in tumorigenesis [35,36]. The dysregulated expression of CYP1B1 was explored between tumor and normal tissues. As a result of the abnormal expression in cancer, CYP1B1 has been defined as a candidate tumor antigen [37]. Interestingly, CYP1B1 has also been exploited as a molecular target for immunotherapy, owing to the restricted expression profile in normal tissues [38]. Differential CYP1B1 expression was also observed in different clinical stages and tumor grades, and a high expression of CYP1B1 was also observed in renal cell carcinoma, which was related to advanced grades and late stages [39]. In vitro and vivo function experiments identified that CYP1B1 could increase tumor progression and metastasis. The results indicated that CYP1B1 was correlated with clinical characteristics in cancer and could be a prognostic predictor in cancer.

In the present study, the infiltration of lymphocyte, immune regulators, CAFs, immune subtype, and molecular subtype was mediated by CYP1B1. By using genetic study of the melatonergic microenvironment across 14 solid tumors, Lv et al. also identified that the melatonin catabolic enzymes, including CYP1B1, were associated with TMB and prognosis [40]. In vitro experiments found that the CYP1B1 expression was significantly correlated with B7-H3 expression in colorectal cancer. Nevertheless, in vivo experiments revealed that HLA-A*0201 could be processed and presented by CYP1B1-specific cytotoxic T lymphocytes (CTLs) [41,42]. The neutrophil infiltration mediated by CYP1B1 in the TME could be reversed by melatonin in vivo. Based on these findings, we speculated that CYP1B1 may regulate the immune cell infiltration in the tumor microenvironment, which could act as a molecular marker for clinical therapy.

TMB, MSI, and neoantigen have been identified as biomarkers for predicting the response of immune checkpoint inhibitors in cancer [43,44]. Our results found that CYP1B1 expression was associated with TMB, MSI, and neoantigen in pan-cancer, especially in LIHC and STAD. ssGESA analysis CYP1B1 was associated with melatonergic and immune-related pathways and therapeutic response. Recent studies showed that CYP1B1 was involved in the drug resistance of tumor cells, such as paclitaxel and docetaxel [45,46,47]. The clinical trial of CYP1B1-directed vaccination identified that patients with solid and hematologic tumors can benefit from anti-CYP1B1 immunity [48]. Thus, targeting CYP1B1 may be a useful way for the development of anticancer treatment.

However, several issues need to be further explored. First, the clinical and immunological characteristics of CYP1B1 in cancer were based on the public database; the roles of CYP1B1 still needs to be further verified by multi-center data. Second, the function of CYP1B1 has been clarified in vitro and vivo, but the function of CYP1B1 knock-down or its inhibition need to be further explored and the potential mechanism of CYP1B1 in TME remains unclear. Third, although the CYP1B1 has promising predictive power for ICI in 11 clinical cohorts, its predictive power still needs to be validated on a larger number of immune cohorts. Finally, the immunological CYP1B1 in pan-cancer needs additional investigation to clarify its function and processes.

## 5. Conclusions

In conclusion, we comprehensively analyzed the clinical and immunological characteristics of CYP1B1 across 33 solid tumors. Our results identified that the dysregulated expression of CYP1B1 was associated with the clinical stage, tumor grade, immune cell infiltration, TMB, MSI, neoantigen, activation of multiple melatonergic and immune-related pathways, and therapeutic resistance. Targeting CYP1B1 might be a promising predictor and molecular target for clinical treatment. 

## Data Availability

The data presented in this study are available in this article and Supplementary Material.

## Supplementary Materials

The following supporting information can be downloaded at: https://www.mdpi.com/article/10.3390/cancers14225641/s1, Figure S1. Forest plots of CYP1B1 expression on the prognosis in cancer. (A) Effect of CYP1B1 on overall survival (OS) in cancer. (B) Effect of CYP1B1 on disease specific survival (DSS) in 33 types of cancers. (C) Effect of CYP1B1 on disease free interval (DFI) in cancer. (D) Effect of CYP1B1 on progression free interval (PFI) in cancer. The red line means risky factor in prognosis, the blue line means protective in prognosis, the grey line means no significance in prognosis. Figure S2. The negative correlation between CYP1B1 expression and tumor grade in LIHC. Figure S3. The correlations between CYP1B1 expression and immunomodulator. (A) The spearman correlations between CYP1B1 expression and immunostimulator in cancer. (B) The spearman correlations between CYP1B1 expression and immunoinhibitor in cancer. The heatmap represents rho value. Red color means positive correlation, blue color means negative correlation. Four associations were showed by dot plots. Figure S4. The correlations between CYP1B1 expression and chemokine and receptor. (A) The spearman correlations between CYP1B1 expression and chemokine in cancer. (B) The spearman correlations between CYP1B1 expression and receptor in cancer. The heatmap represents rho value. Red color means positive correlation, blue color means negative correlation. Four associations were showed by dot plots. Figure S5. The IC50 between high and low CYP1B1 expression in normal tissue.
