# Supplementary figures and images for: CYP1B1: A Novel Molecular Biomarker Predicts Molecular Subtype, Tumor Microenvironment, and Immune Response in 33 Cancers
Source: Cancers (Basel). 2022 Nov 17;14(22):5641. doi: 10.3390/cancers14225641 (PMC9688555; doi:10.3390/cancers14225641)

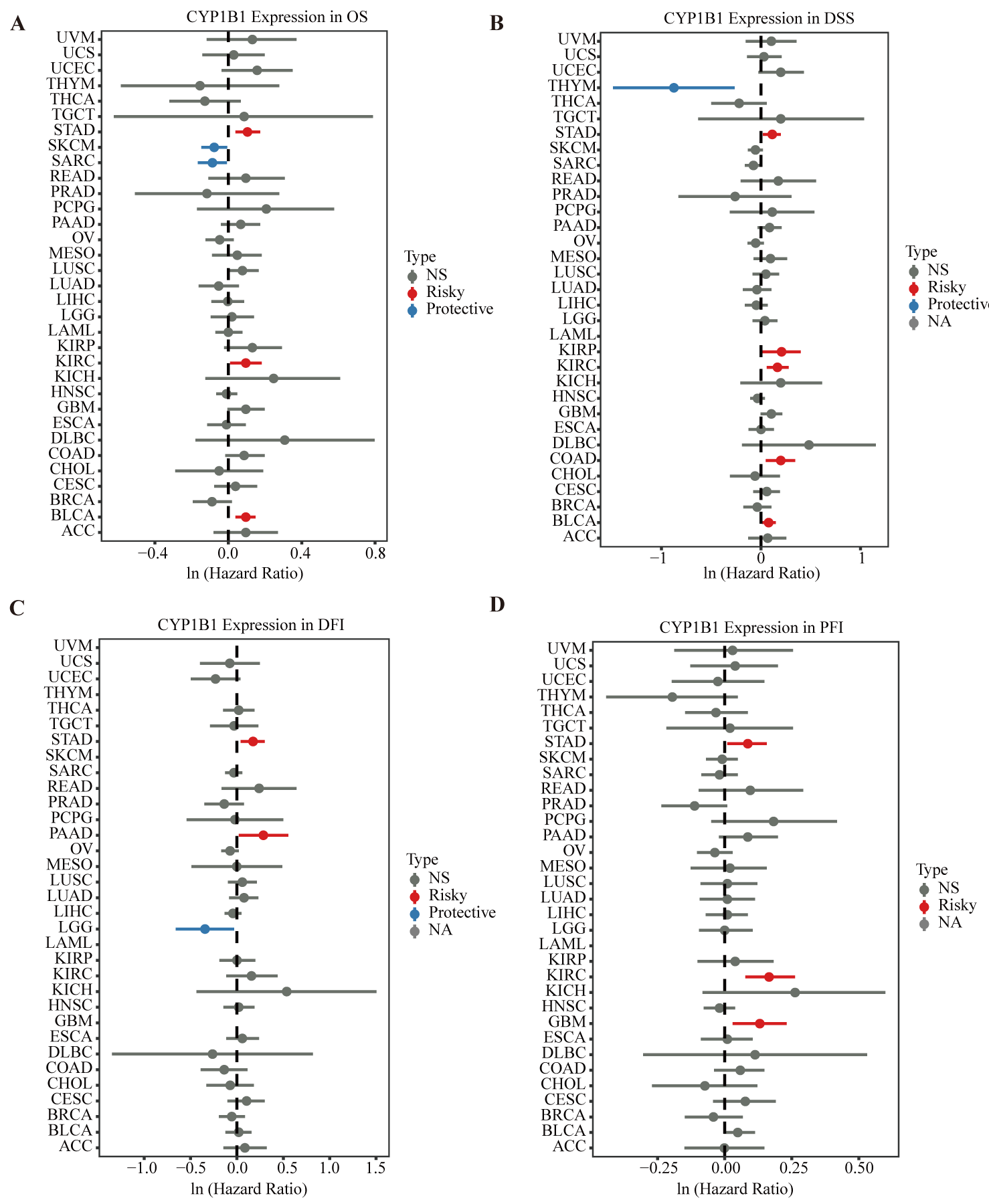

Supplement: Supplementary file 1 [file cancers-14-05641-s001.zip › Figure-S1.tif]

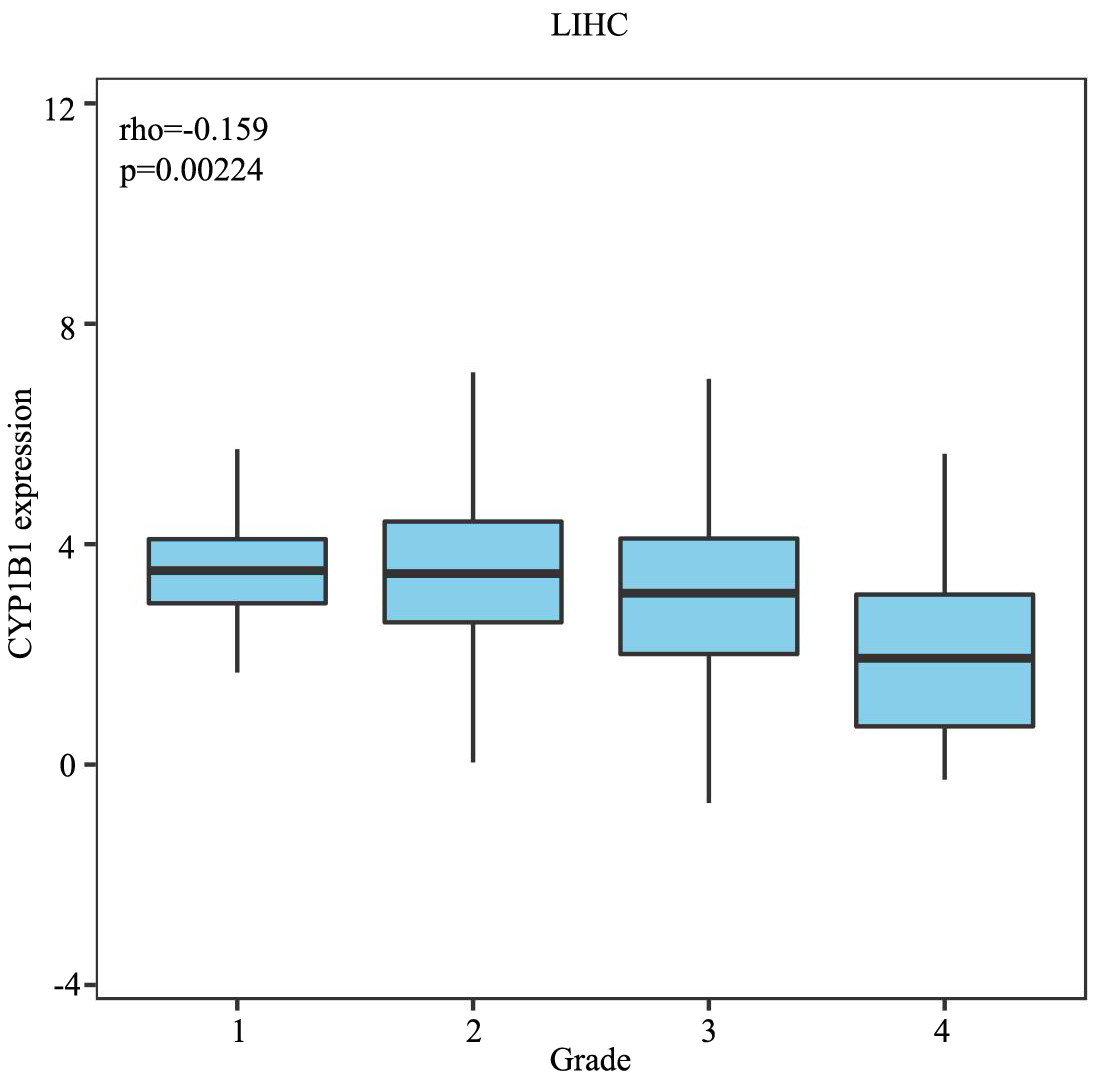

Supplement: Supplementary file 1 [file cancers-14-05641-s001.zip › Figure-S2.tif]

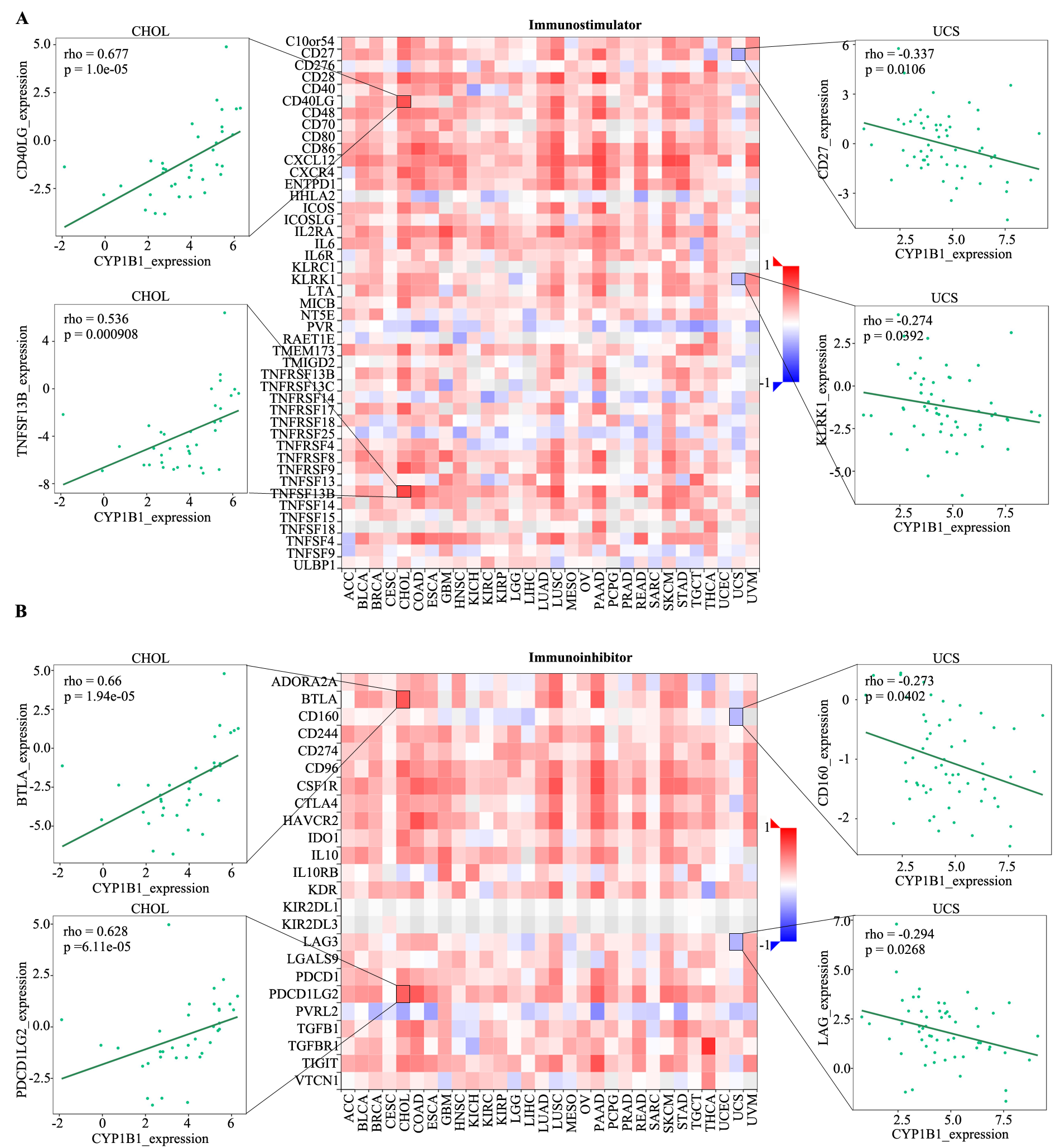

Supplement: Supplementary file 1 [file cancers-14-05641-s001.zip › Figure-S3.tif]

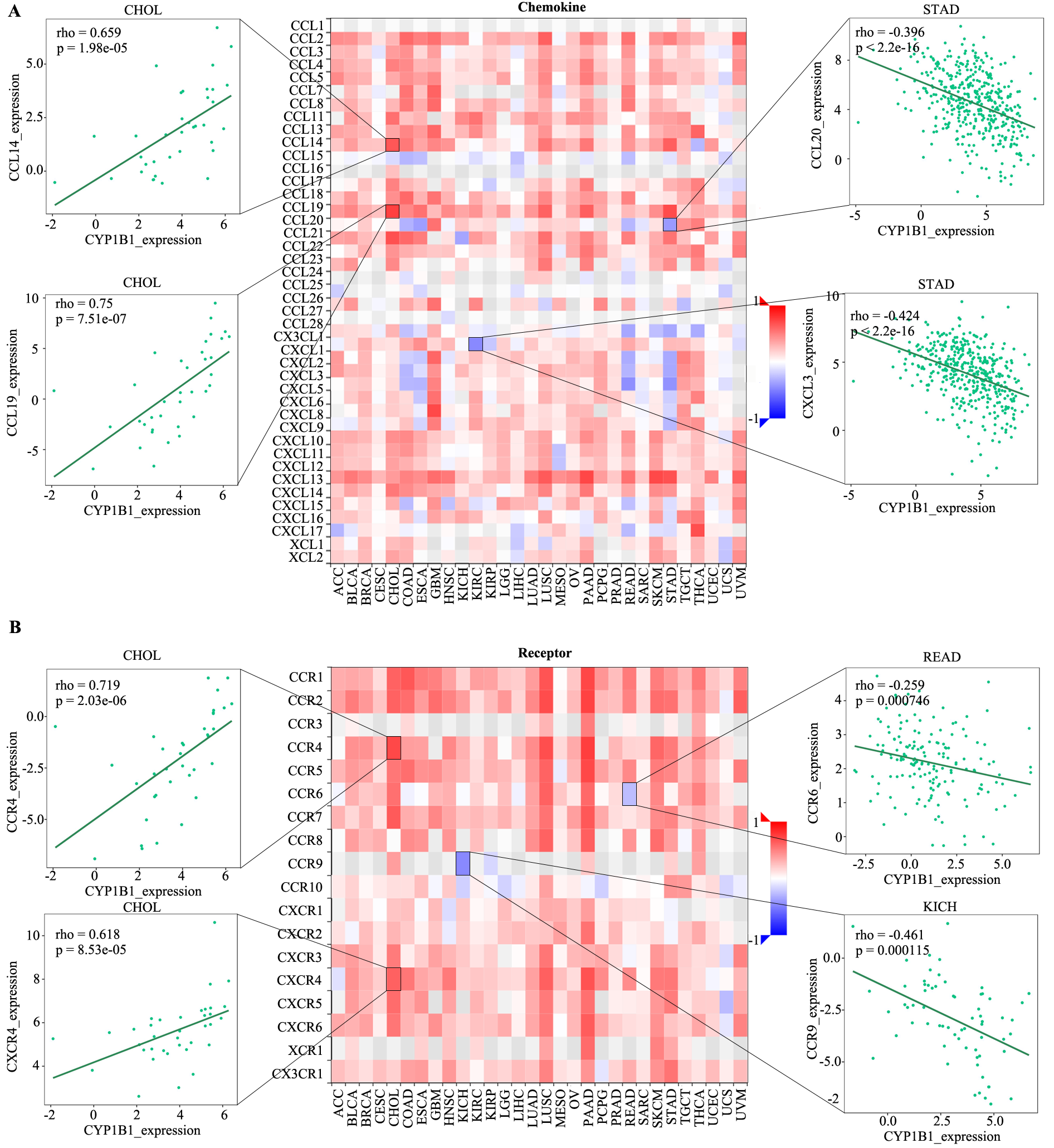

Supplement: Supplementary file 1 [file cancers-14-05641-s001.zip › Figure-S4.tif]

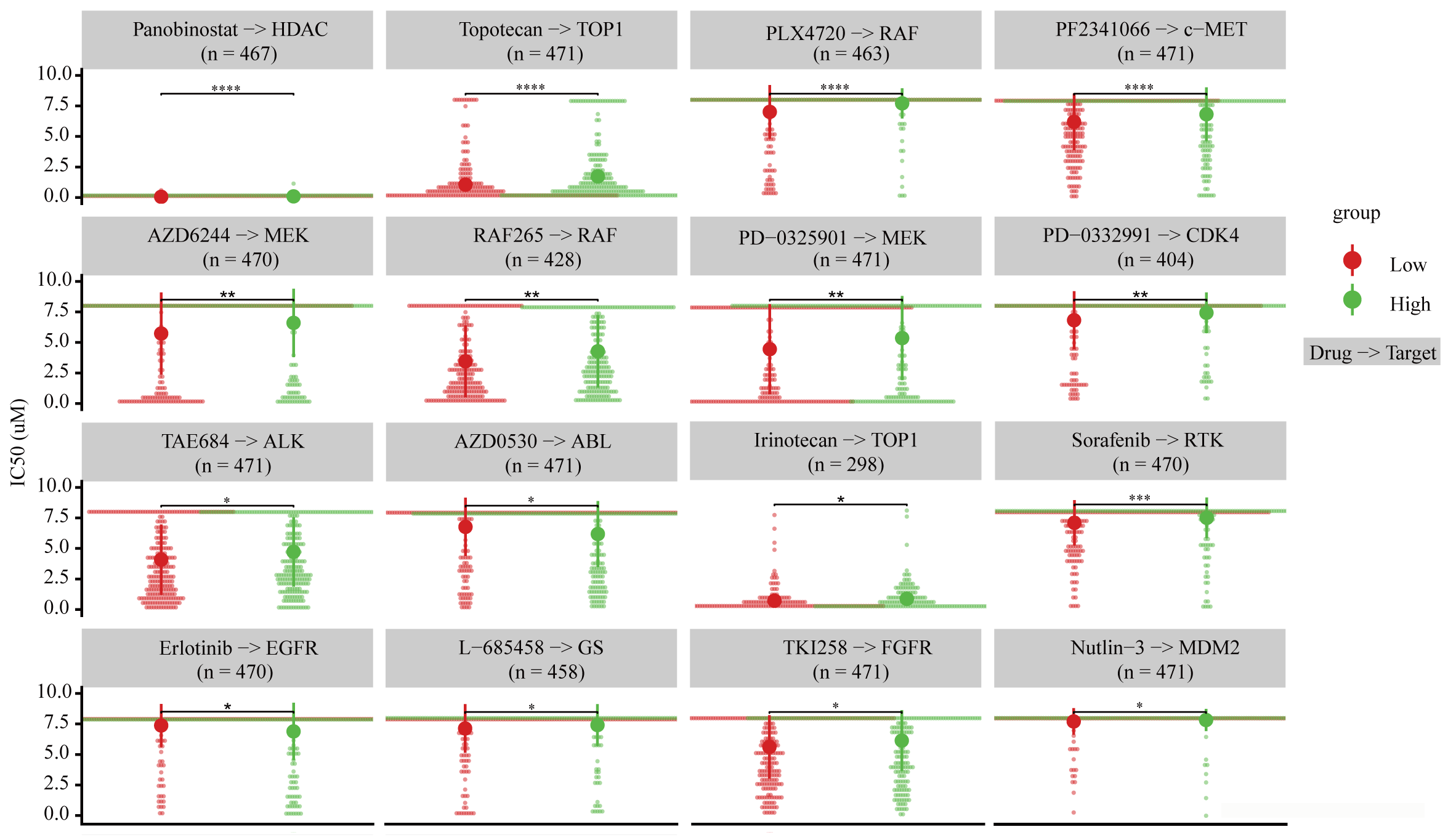

Supplement: Supplementary file 1 [file cancers-14-05641-s001.zip › Figure-S5.tif]
